# Sea Marks

**Published:** 1986-10

**Authors:** H. G. Mather


**SEAMARKS; THEIR HISTORY AND DEVELOPMENT**

**John Naish**
**Stanford Maritime, London. pp. 192. £12.95**

This is the first comprehensive history and survey of buoys, landmarks, lighthouses and other guides to navigation. It represents an enormous amount of research by the author, the well known retired Bristol Physician, John Naish. That it gave him a lot of pleasure comes through in the text, which is written in a way that is both authoritative and interesting, even to a landlubber. He traces the history from the third century B.C. to modern times, and has collected excellent illustrations. The author has had a lifelong interest in sailing in small boats, naval vessels and cruising yachts, so he really appreciates the importance of seamarks. Anyone who sails will want to read this book which brings the subject right up to date by discussing radar and the discipline of traffic regulation in the Channel. As a maritime nation, we neglect seamarks at our peril, and I was reminded of the book last month when I saw the Trinity House vessel, Winston Churchill, servicing the West and East Narrows buoys in Falmouth harbour.

H. G. Mather

